# Why Does Not Nanotechnology Go Green? Bioprocess Simulation and Economics for Bacterial-Origin Magnetite Nanoparticles

**DOI:** 10.3389/fmicb.2021.718232

**Published:** 2021-08-20

**Authors:** Tarcisio Correa, Rogério Presciliano, Fernanda Abreu

**Affiliations:** Laboratório de Biologia Celular e Magnetotaxia, Instituto de Microbiologia Paulo de Góes, Universidade Federal do Rio de Janeiro, Rio de Janeiro, Brazil

**Keywords:** magnetotactic bacteria, magnetosomes, magnetic nanoparticles, biominerals, techno-economic analysis, process simulation, clean production

## Abstract

Nanotechnological developments, including fabrication and use of magnetic nanomaterials, are growing at a fast pace. Magnetic nanoparticles are exciting tools for use in healthcare, biological sensors, and environmental remediation. Due to better control over final-product characteristics and cleaner production, biogenic nanomagnets are preferable over synthetic ones for technological use. In this sense, the technical requirements and economic factors for setting up industrial production of magnetotactic bacteria (MTB)-derived nanomagnets were studied in the present work. Magnetite fabrication costs in a single-stage fed-batch and a semicontinuous process were US$ 10,372 and US$ 11,169 per kilogram, respectively. Depending on the variations of the production process, the minimum selling price for biogenic nanomagnets ranged between US$ 21 and US$ 120 per gram. Because these prices are consistently below commercial values for synthetic nanoparticles, we suggest that microbial production is competitive and constitutes an attractive alternative for a greener manufacturing of magnetic nanoparticles nanotools with versatile applicability.

## Introduction

The global nanotechnology market is forecast to reach US$ 173 billion in 2025, with a large share of this growth boosted by environmental and biomedical sectors (Gharailou, 2019). One of the significant pillars of nanotechnology relies on magnetic nanoparticles. Revenues generated from iron oxide nanoparticles expand 11% annually, with a projection of US$ 5 billion in 2023 (Nano-Powder Factory, 2020). Clean manufacturing of high-quality nanoparticles must be achieved to sustain such growth and supply the increasing demand for innovative products and processes.

The magnetic-stimuli responsive character of iron-oxide nanoparticles enables their use in environmental remediation, biosensing, and healthcare (Kudr et al., 2017; Jiang et al., 2018). Explored roles of magnetic nanoparticles include but are not limited to oil and heavy metal adsorptive materials, drug delivery vectors, magnetic resonance contrast agents, theranostic agents for cancers and pollution, and pathogen detectors (Kudr et al., 2017; Jiang et al., 2018). However, in real-world applications, these materials require large-scale processes capable of delivering nanomagnets with controlled and reproducible characteristics (Tartaj et al., 2019).

An overwhelming number of published and patented methods were developed for obtaining magnetic nanoparticles through physical, chemical, and biotechnological routes (Krishnan et al., 2016; Tartaj et al., 2019; Abreu et al., 2020a). Within the latter category, magnetotactic bacteria (MTB) constitute the primary microbial magnetic nanoparticle source (Iravani and Varma, 2020). MTB are present in basically all aquatic environments, where they use chains of magnetic organelles as a compass to migrate in a directionally oriented manner (Abreu et al., 2020b). These magnetic structures, or magnetosomes, can be extracted from MTB cells and used as biological-origin magnetic nanoparticles (BMNs).

The intracellular formation of BMNs is a complex and genetically controlled biomineralization process (Correa et al., 2021). Genes responsible for BMNs biomineralization are clustered within the bacterial genome (Abreu et al., 2020b). Steps are iron capture from the environment and precipitation into iron mineral inside intracytoplasmic projections or vesicles formed from the internal bacterial membrane (Correa et al., 2021). The iron mineral composition, either magnetite (Fe_3_O_4_) or greigite (Fe_3_S_4_), generally depends on the MTB species (Abreu et al., 2020b; Correa et al., 2021). Due to the gene-level orchestrated biochemistry underlying BMNs formation, the mineral nanocrystals usually have narrow size dispersibility, consistent with a stable single magnetic domain, precise particle shape, and crystalline purity (Vargas et al., 2018; Abreu et al., 2020b). As iron biomineralization occurs within vesicles, each magnetic nanocrystal retains the membrane envelope after physical isolation processes (Vargas et al., 2018).

**FIGURE 1:**
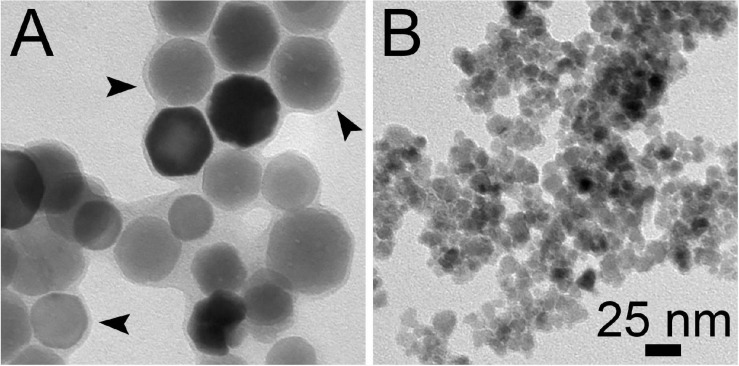
Transmission electron microscopy images of BMNs isolated from a *Magnetospirillum gryphiswaldense* strain MSR-1 **(A)** and synthetic iron oxide nanoparticles **(B)** prepared by co-precipitation (Santos et al., 2018). Arrowheads indicate external BMN membrane, which is retained after isolation process.

Applications for various technological purposes have been proposed for purified BMNs (Vargas et al., 2018). Most of these studies make use of magnetite BMNs isolated from MTB affiliated to the *Magnetospirillum* genus, which are cubooctahedral in morphology and whose diameters range between 30 and 40 nm (Figure 1; Pósfai et al., 2013). Besides phospholipids, proteins involved in biomineralization are also present in BMNs membranes. These proteins are the basis for surface functionalization of BMNs because they can be either chemically modified for insertion of drugs or antibodies or genetically fused with enzymes, antibodies, receptors, binding proteins, and stimuli-responsive peptides (Vargas et al., 2018). After functionalization, BMNs can integrate vaccine and drug formulations (Tang et al., 2012; Geng et al., 2019), immunomagnetic sensors for food pathogens (Xu et al., 2019; Sannigrahi et al., 2020), and cell sorting nanotools (Yoshino et al., 2008). Owing to heat generation by nanomagnets exposed to oscillating magnetic fields, BMNs have also been used in *in vivo* hyperthermal tumor inhibition (Alphandéry et al., 2019). In the environmental area, BMNs support recoverable and reusable catalysts for pesticide degradation (Ginet et al., 2011) and clean industrial processes (Honda et al., 2015). BMNs could also be used in the generation of clean energy (Smit et al., 2018).

The production of metallic nanoparticles by microbial factories, including MTB, has been extensively reviewed (Ahmad et al., 2019; Grasso et al., 2020; Iravani and Varma, 2020). One consensus is that biological nanomanufacturing of these materials is environmentally friendly because these processes do not usually rely on aggressive chemicals. For this reason, the mass production of nanomagnets through MTB-based bioprocessing is in strong agreement with UN’s Sustainable Development Goal 9 to “upgrade infrastructure and retrofit industries to make them sustainable, with […] greater adoption of clean and environmentally sound technologies and industrial processes” (United Nations (UN), 2020).

Multiple studies have been dedicated to increasing BMNs throughput in bioreactors (3–70 L) MTB cultures (Silva et al., 2013; Basit et al., 2020; Berny et al., 2020). The main challenges concerning the cultivation of MTB in large volumes are the microaerophilic metabolism of this group, relatively slow growth rates, and specific nutritional requirements, in addition to BMNs yields in the order of mg/L (Ali et al., 2017; Basit et al., 2020). Strategies like optimizing oxygen supply and balanced nutrient injection, have been attempted to address such hurdles and led to improved production yields (Liu et al., 2010; Zhang et al., 2011; Fernández-Castané et al., 2018). However, modeling production on a pilot and industrial scale is yet to be done. Thus, given all the applicability and green production of BMNs, we have performed bioprocess simulation and approximate economic assessment of BMNs production at industrial scales. The techno-economic analysis may help identify process opportunities and challenges and is necessary before upgrading BMNs production to industrial levels.

## Materials and Methods

### Production Scale

**FIGURE 2:**
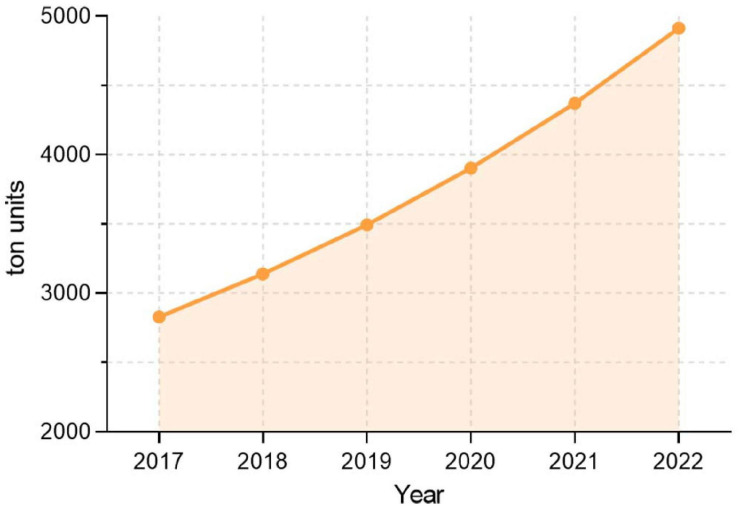
Growth in international consumption of iron-oxide nanoparticles over the years of 2017–2022. Data from Nano-Powder Factory (2020).

Our selected process throughput has been calculated to meet the demand of iron oxide nanoparticles of Latin America in environmental and healthcare industries. The calculation is summarized in Supplementary Table 1 and was based on the International consumption of iron oxide nanoparticles (Figure 2; Nano-Powder Factory, 2020) and the number of registered nanoproducts (Supplementary Figure 1; StatNano, 2020).

### Design Basis

The simulated plant comprises three sections: an inoculum train, a fermentation section, and a downstream BMN recovery section. The magnetotactic spirillum *Magnetospirillum gryphiswaldense* strain MSR-1 was chosen as the BMNs-producing microorganism. MSR-1 cells are microaerophilic and produce chains of cubooctahedral magnetite BMNs with a ∼35 nm diameter (Schüler et al., 2020). For transmission electron microscopy images displayed here, cells were deposited on formvar-coated copper grids and observed on a FEI Morgagni transmission electron microscope (Hillsboro, OR, United States) operating at 80 kV.

### Modeling and Simulation Software

SuperPro Designer v9.0 (Intelligen, United States) was used for process modeling and simulation in both single-stage fed-batch and semicontinuous scenarios, as well as the proposed variations considered to sensitivity analyses. Information used for process design and data input to simulation software is detailed in the next subsections.

### Economic Data and Calculation

The economic data were selected for a plant located in the state of Rio de Janeiro, Brazil. Capital and operational costs, including equipment and fabrication cost, were calculated using built-in models of SuperPro Designer, which are based on the methodology described in Supplementary Tables 2, 3. Minimum selling prices (MSP) were determined as stipulated by Seider et al. (2016) for a fixed payback time of 5 years. For that, we performed multiple economic calculations on SuperPro Designer using different hypothetical selling prices (US$ 30–120 thousand/kg Fe_3_O_4_). Materials, utilities, and financial data collected for this study are detailed in Supplementary Tables 4–9.

### Upstream Section

The inoculum train was composed of three consecutive seed bioreactors with an expansion factor of 10 up to the volume of the main bioreactor (see section “Fermentation section”). The medium used in this section was the same as the fermentation medium.

### Fermentation Section

Fermentation was assumed to be conducted in a fed-batch, as summarized in Table 1. A medium preparation tank was allocated for the preparation of both fermentation and feeding media. Air compression was required for low-rate oxygen supplying (0.002–0.003 vvm) during cell growth. The principal bioreactor was designed to a total volume of 29 m^3^, which must contain the initial fermentation medium (∼15 m^3^) and additional feeding volumes without exceeding 80% of the vessel capacity. The initial pH of the fermentation medium was set to the range 6.8–7.0 and temperature should be kept at 30°C.

**TABLE 1:** Parameters considered for simulation–fermentation section (base case).

**Parameter**	**Value**	**References**
Fermentation time	42 h	Zhang et al., 2011
Fermentation temperature	30°C	Zhang et al., 2011
pH	6.8–7.0	Zhang et al., 2011
Growth rate (μ)	0.01	Zhang et al., 2011
Aeration rate	0.003 vvm	Zhang et al., 2011
Production of Fe_3_O_4_	250 mg/L	Zhang et al., 2011
Volume of the main fermenter	29 m^3^	Modeled
Maximum working volume	80%	Modeled
Vessel material	Stainless steel 316	Assumed

The selected fermentation designs, single-stage and semicontinuous, were based on the process described by Zhang et al. (2011) for having the highest reported magnetite yield in literature. The single-stage process was a fed-batch carried in a fermentation medium (Zhang et al., 2011). The feeding medium contained an iron source for magnetite synthesis (iron chloride) and lactic acid as the main carbon source, among other nutrients. The feeding regime was based on the pH change in culture media during cell growth. As cell growth leads to alkalinization in the culture medium, appropriate automatic pH control is necessary. Due to the high concentration of lactic acid, which causes a pH of 2.5–3.0, the feeding medium was supplied to fermentation media in response to increases in pH.

In semicontinuous operation, cultivation is carried out for 40–44 h (first stage) and then, 90% of the fermentation medium is removed to the downstream section (Zhang et al., 2011). Afterward, the original fermentation volume is restored with the addition of sterile fermentation media to the remaining 10% first stage medium. The second stage is started as a fed-batch in the same manner as the first one.

The highest yield of BMNs reported for a large-scale process (356.52 mg/L) was reached in a single-stage fermentation in a 42-L bioreactor for 44 h (Zhang et al., 2011). Nevertheless, this value was associated with a single fermentation and, to our knowledge, such yield has not been reproduced in literature. Other yields reported by the same paper were lower (225–280 mg/L) when cells were cultured in 7.5-L for 40–44 h under an identical fed-batch regime. For this reason, we assumed a yield of 250 mg/L within 42 h for our base-case simulation. Jajan et al. (2019) reported the production of 186 mg/L of BMNs, which was close to our assumed value and supports a more realistic simulation. The specific cell growth (μ) was calculated from Zhang et al. (2011) and equals 0.10.

The fermentation stoichiometry and molecular formula for *Ms. gryphiswaldense* strain MSR-1 were described in Naresh et al. (2012). The global equation for bacterial growth is:

352CH3O6+333.6NO+3-133NH+4+6.46O2

+1.25Fe→3+718.4MTB+591.3HO2

$$+314CO{}_{2}$$

While the molecular formula for the magnetotactic bacterium (MTB) is CH_2.0__6_O_0.1__3_N_0_._2__8_Fe_0.00174_.

### Downstream Section

Biological-origin magnetic nanoparticles (BMNs) extraction was based on Guo et al. (2011) and Rosenfeldt et al. (2020). The assumed simulation parameters for this section are summarized in Table 2. Detailed information on the modeling of the downstream section is available in Supplementary Material. Fermented medium was transferred from the bioreactor vessel to a high-pressure homogenizer for cell crushing. The cell lysate was, then, eluted through a magnetic separation column (MSC), whose design is comprised of an aluminum column with a magnetizable matrix. The matrix was made of 2-mm diameter stainless steel beads that can be magnetized by placing two neodymium plates externally onto the column. MSC design was sketched in Supplementary Figure 2 and its costing details are described in Supplementary Table 7. During separation, the magnetic concentrate was washed with 4 M urea for the removal of residual proteins from cell lysate. The magnetic concentrate was further purified by disk-stack centrifugation, which concentrates 90% of BMNs. As in Rosenfeldt et al. (2020), sucrose syrup was mixed to the magnetic concentrate before centrifugation for retention of cell residues. Afterward, the concentrate was eluted in a second MSC, for the final removal of impurities. The final product was a magnetic colloid containing 1 mg/mL BMNs suspended in a phosphate buffer.

**TABLE 2:** Parameters considered for simulation–downstream section (base case).

**Parameter**	**Value**	**References**
Number of passes at high pressure homogenizer	4	Rosenfeldt et al., 2020
Magnetic separation column (MSC) matrix	Stainless steel 2-mm beads	Adapted from Guo et al. (2011)
Binding capacity of MSC matrix	85%	Estimated from Guo et al. (2011) and Rosenfeldt et al. (2020)
MSC material	Aluminum + neodymium plates	Assumed
MSC flowrate	3 bed volumes/h	Assumed
Centrifuge sedimentation efficiency	90%	Assumed

### Sensitivity Analyses

The influence of changes in economic and operational conditions was studied for both fermentation processes by altering each parameter within modeling software and updating material balance and economic calculations.

## Results

**FIGURE 3:**
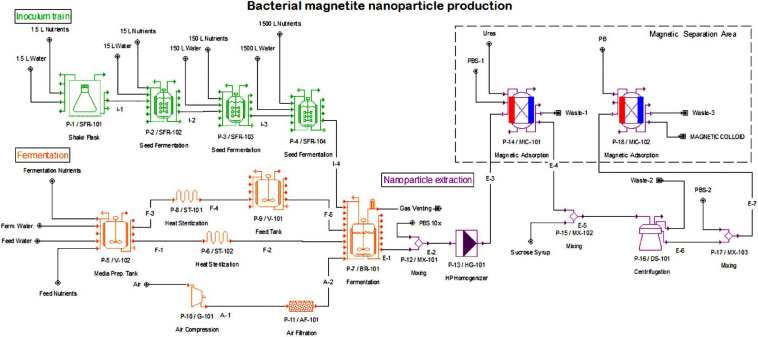
Process flowsheet for the biomanufacturing of BMNs. The flowsheet depicts unit operations and material flows simulated in this work. The overall process is divided into three sections: the inoculum preparation train (in green) with four sequential scale-up steps, 3L (P-1), 30L (P-2), 300L (P-3), and 3000L (P-4), the fermentation (P-7), including media preparation (P-5), sterilization (P-6 and P-8), storage (P-9), air compression (P-10) and filtration (P-11) (in orange) and the downstream BMN extraction, including operations for cell lysis (P-12 and P-13), centrifugation (P-15 and P-16), and magnetic separation (P-14, P-17, and P-18) (in purple).

The plant capacity assumed from the market study calculations is 640 kg BMNs per year in our base case. From the assumptions described throughout the methodology section, our process flowsheet was designed in SuperPro and is displayed in Figure 3. Plant and operating costs are summarized in Table 3, as well as batch scheduling information. From the initial project investment, approximately 20% is for equipment purchase in both single-stage and semicontinuous. The remaining project investment is directed to plant engineering and construction, including equipment installation, electrical setup, and piping. The main bioreactor of the fermentation sector represents 30.6% of all equipment cost in a single-stage setting (Supplementary Table 8) and 38.2% in semicontinuous (Supplementary Table 9).

**TABLE 3:** Overall bioprocess parameters and economic evaluation summary.

	**Single stage**	**Semicontinuous**
Annual operating time (h)	7,200	
Recipe batch time (h)	161.7	193.1
Recipe cycle time (h)	46.67	85.75
Number of batches per year	151	82
Annual Fe_3_O_4_ throughput (kg)	640	
Capital investment	52.11	79.86
Total plant cost (US$ millions)	43.03	66.02
Equipment cost (US$ millions)	8.90	12.89
Operating cost (US$ millions/year)	6.64	7.15
Unit production cost (US$/kg Fe_3_O_4_)	10,372	11,169

**FIGURE 4:**
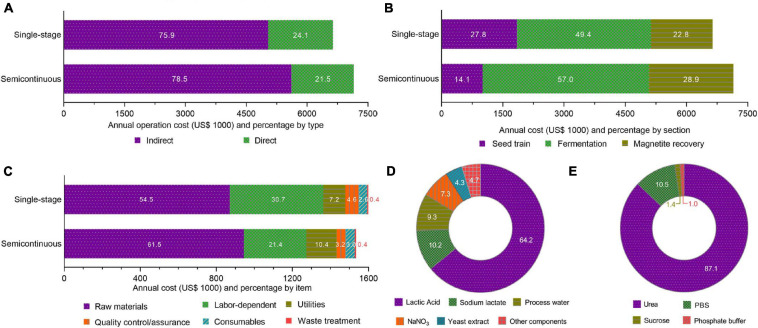
Annual operating costs composition breakdowns. Single-stage and semicontinuous cultivation cost contributions from direct and indirect costs **(A)** and process sections **(B)**. Direct operating costs breakdown showing cost types **(C)**. Material costs compositions from fermentation **(D)** and downstream sections **(E)**.

Operating costs breakdowns for single stage (US$ 6.64 million/year) and semicontinuous (US$ 7.15 million/year) modes are summarized in Figure 4. However, for both cases, indirect operating costs (i.e., maintenance, equipment depreciation, local taxes, etc.) are 3–4 times higher (Figure 4A) than direct costs (i.e., raw material, labor, quality control, etc.). In single-stage, about half the operating costs are related to the fermentation section, whereas this share increases at 7% in semicontinuous (Figure 4B). In both operation modes, material costs represent more than half the direct operating costs (Figure 4C). Although total operating costs are higher in semicontinuous mode, direct operating costs are slightly higher in a single stage. This is caused by a 9% growth in labor-dependent cost in a single stage. Lactic acid and urea represent the highest share of material costs in fermentation (64%) and downstream (87%) sections, respectively (Figures 4D,E).

**FIGURE 5:**
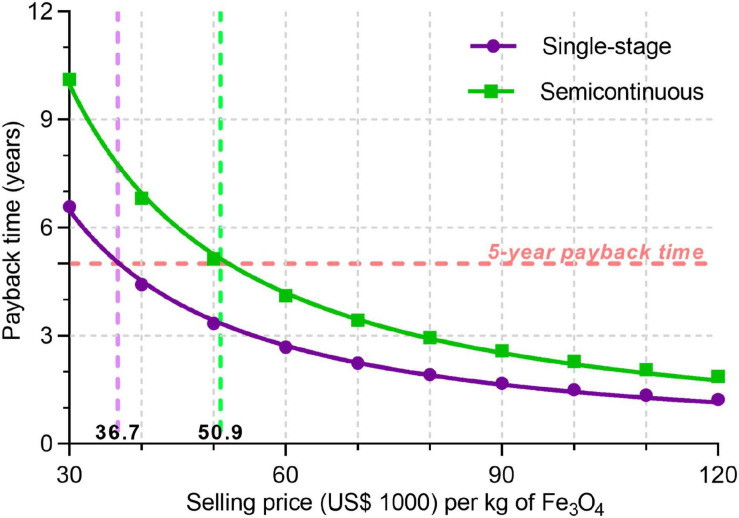
Correlation between selling prices of BMNs and the investment payback time for each bioprocess. The salmon horizontal dashed line indicates the 5-year payback time limit for which MSPs are determined. Vertical dashed lines indicate MSPs for single-stage (purple line, US$ 36.7 thousand) and semicontinuous (green line, US$ 50.9 thousand) processes.

**FIGURE 6:**
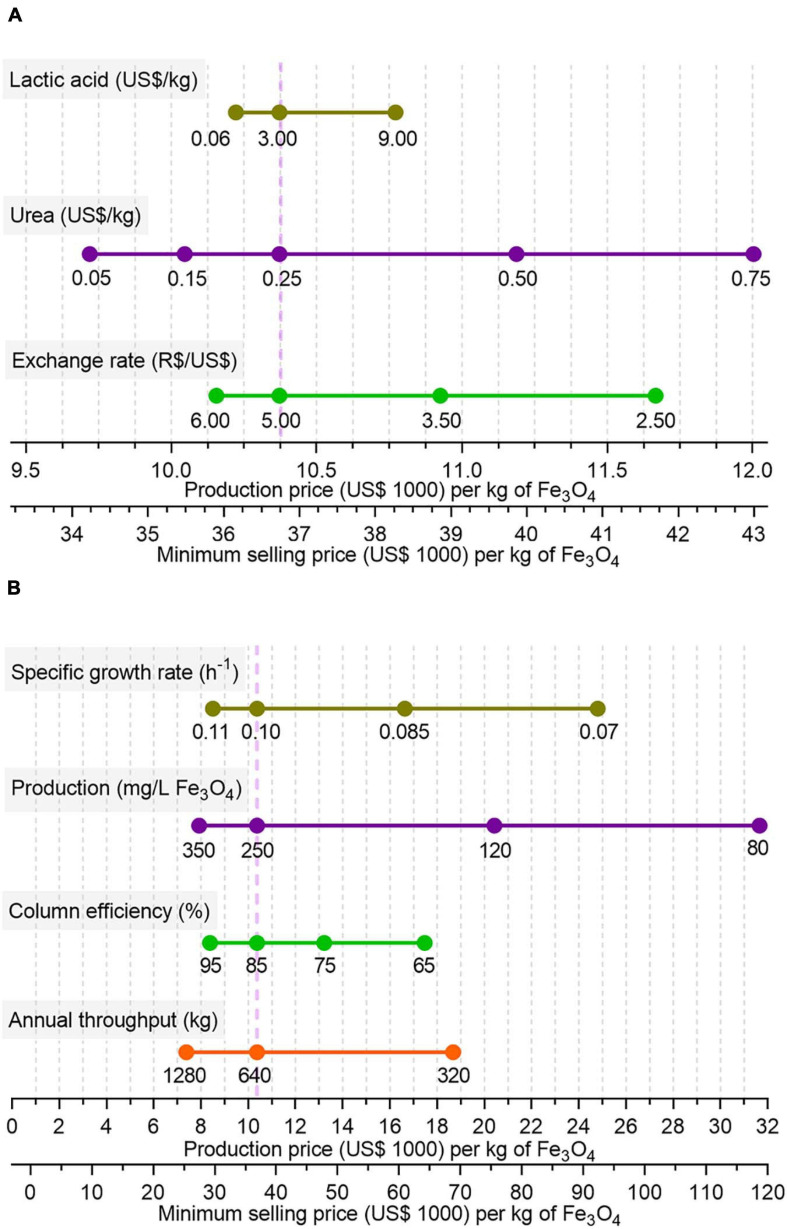
Sensitivity analyses showing effects of variations in market **(A)** and bioprocess-related parameters **(B)** on unitary production costs and minimum selling prices (MSP) for the single-stage process, when one of those individual parameters oscillate from the base-case (purple dashed vertical line). The results of variations in purchase price of two important feedstocks (lactic acid and urea) and fluctuations of the exchange ratio on the final production price are analyzed in panel **(A)**. The effects of specific cell growth rate of *Ms. gryphiswaldense* MSR-1 in fermentation tanks, as well as its magnetite production rate, are analyzed in panel **(B)**. The efficiency in BMN extraction by the MSCs and the plant annual throughput capacity, in terms of total produced magnetite, are also assessed in panel **(B)**. The purple dashed vertical line indicates base-case scenario whose parameters are described in Tables 1, 2.

Considering the scenarios listed in Table 1, MSPs of BMNs are US$ 36.7 and 50.9 thousand/kg Fe_3_O_4_ for single-stage and semicontinuous processes, respectively (Figure 5). Sensitivity analyses indicate economic parameters (material costs, dollar/real exchange rates) seem to have a more slight effect on operating costs and MSP of BMNs than operational and microbial parameters (Figure 6 and Supplementary Figure 3). For example, while a variation from US$ 0.05 to 0.75 in the urea purchasing price caused the operating costs to fall between US$ 9,700 and 12,000/kg (Figure 6A and Supplementary Figure 3A), a magnetite yield of 80 mg/mL might raise operating costs to US$ 32,000 (Figure 6B and Supplementary Figure 3B).

## Discussion

In recent years, Latin American countries have focused on nanotechnology as a stimulus for economic growth through the production of nanometric materials and incorporation of nanotechnological tools into industrial processes and products (StatNano, 2016). Despite the share of registered nanotechnological products in the region is still small (2.4%), programs like the Brazilian Initiative for Nanotechnology (Ministério da Ciência, Tecnologia, Inovações e Comunicações (MCTI), 2019) and the Argentine Foundation of Nanotechnology (Fundación Argentina de Nanotecnología (FAN), 2020) are examples of government-led support for an expansion in the nanotechnological industry.

In our assessment, indirect operating costs represented 76–79% of all operating costs due to facility-related expenditures. Usually, facility-related costs represent 10–70% of operating costs while direct costs account for 50% of the total (Harrison et al., 2015). In the Brazilian market, prices of raw material from local suppliers, utilities, and salaries tend to be lowered in comparison to US prices due to the exchange rate in recent years (US$ 1 = R$ 5.20 in June, 2020). In a process simulation for glucosidase production (da Gama-Ferreira et al., 2018), a low direct cost accounting (23–25%) was also attributed to Brazilian market conditions and real-dollar exchange rate. Moreover, as our bioprocess requires large equipment volumes, especially in the fermentation section (Supplementary Tables 8, 9), maintenance and depreciation costs are substantially higher than low-cost bacterial growth media components (Supplementary Table 4).

While the fermentation section spends the higher fraction of operating costs, BMN recovery accounted for only 22–29%. The magnetic nature of magnetite crystals along with its high density (5.18 g/cm^3^) facilitates their separation from cell lysate during the downstream section. These material properties have facilitated the development of continuous, large-scale strategies for BMN recovery (Guo et al., 2011; Rosenfeldt et al., 2020). The simple design of MSC (Supplementary Figure 2) is associated with low purchase and installation costs (Supplementary Tables 7–9). Additionally, the durability of the magnetizable separation matrix reduces the necessity of operational intervention and maintenance within the downstream section.

The increase of 7.7% in the cost for the fabrication of one ton of BMNs by semicontinuous operation in relation to single-stage (Figure 4) is attributed to an over 50% decrease in magnetite yields during the second stage (Zhang et al., 2011). This reduction is probably because a deacceleration in bacterial growth is observed when cultivation occurs in oxygen levels under 1% and high iron concentrations (Sun et al., 2008; Liu et al., 2010), in which magnetosome synthesis is favored metabolically (Wang et al., 2016). Furthermore, as shown in Figure 4A the higher operating costs in semicontinuous are driven by the greater indirect operating costs. The total medium volume per batch in semicontinuous cultivation is about twice that in a single stage. This increase leads to a necessity of larger and/or multiple pieces of equipment for medium preparation, fermentation, and cell lysis (Supplementary Table 9). Consequently, an escalation in equipment-related costs is observed.

On the other hand, the observed increase in direct operating costs in a single stage (Figure 4C) is caused by a growth in labor-related demand. This additional demand is explained by a more frequent necessity of operator-supervised inoculation and cleaning-in-place procedures within a year. In semicontinuous cultivation, batch and cycle times are longer because two fermentation stages are conducted from a single inoculation. Therefore, semicontinuous operation demands fewer manual steps per mass of fabricated BMNs than those for a single stage. Our sensitivity analyses (Figure 6 and Supplementary Figure 3) indicate that the proposed bioprocess costs and selling prices are robust to even drastic variations in raw material prices and the dollar-real exchange rate. The minor contribution of materials and utilities to fabrication costs seems to buffer the effect of such variations. Nevertheless, microbial growth and magnetite production have more pronounced pressure on process prices. A 50% reduction in magnetite production doubles production costs and MSP and a 30% reduction in growth rate increases these costs by a factor of 2.5. Such instability might have important implications in process upscaling projects because dramatic differences in the production of magnetite are reported for MTB cultivation processes. For example, while Zhang et al. (2011) reported a 356 mg/L yield, Liu et al. (2010), which used a similar pH-coupled feeding strategy, achieved a magnetite production of only 83.2 mg/L.

Understanding process cost perturbations due to cultures yields are crucial for process scale-up. Yields obtained in small-scale are not always reproduced in plant scale due to factors, including shear forces, medium homogeneity, and gas diffusion (Mahdinia et al., 2019). As, to our knowledge, MTB cultivation has only been performed in bioreactors of up to 70 L (Berny et al., 2020), there is still a lack of information on process performance in m^3^-scales for industrial production. For example, the maintenance of proper microaerophilic or anaerobic conditions for biomineralization, which requires sophisticated control strategies in bench-scale cultivation (Sun et al., 2008; Liu et al., 2010), can be more challenging in larger volumes and might directly affect BMN production. Thus, previous knowledge on the sensitivity to magnetite yields is fundamental for risk assessment associated with processing scale-up. Recently, a molecular engineering tool was used to increase BMN production in *Magnetospirillum magneticum* strain AMB-1 (Arakaki et al., 2020). The technique consisted of inserting a plasmid containing a gene region responsible for magnetic BMN synthesis. The transformed AMB-1 cells were able to double their intracellular BMNs number from 21.9 ± 3.5 to 44.4 ± 9.1. In a 10-L fermentation, a 14.6 increase in BMN production was observed for the transformed strain in relation to the wild type. This achievement provides a powerful tool to keep high-producing MTB cells in the industry without affecting process economics.

The efficiency of MSC in recovering BMNs also exert a considerable influence on process economics. An efficiency of 65% might raise the production cost to US$ 18,000/kg (Figure 6B). As the performance of the cubic-meter scale, MSC might be significantly different from the bench-scale apparatus, possible variations in separation must be evaluated. In our simulation, the reduction in the diameter of magnetizable beads from our reference work might prevent reductions in magnetic separation capacity by increasing the matrix surface area.

Alternative annual process throughputs were also investigated in the sensitivity analysis. During project development, the production demand might expand to other regions or industrial sectors or be restricted to a more local market. Interestingly, duplication in the plant processing capacity leads to a 25% drop in production price but when it is half our base case production price and MSP reaches almost US$ 19,000/kg (Figure 6B).

**FIGURE 7:**
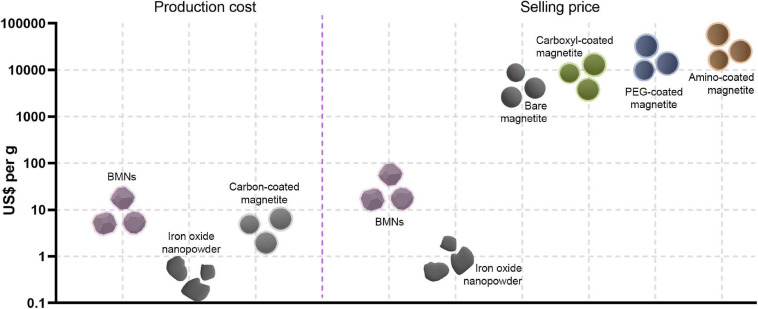
Comparison between production costs (left of dashed vertical line) and market selling prices (right of vertical dashed line) of BMNs and chemically manufactured magnetite nanoparticles, with and without coating. For BMNs, we considered MSPs for market prices. For commercial magnetic nanoparticles, we considered production prices reported by Augusto et al. (2020). For details on the characteristics and suppliers of commercial magnetic nanoparticles, please refer to Supplementary Table 11.

Our base-case production cost (US$ 10,372/kg) is still higher than those for magnetite nanoparticles produced by chemical processes (Figure 7 and Table 4). Augusto et al. (2020) reported a technical-economic analysis for the fabrication of bare magnetite nanoparticles produced by co-precipitation and carbon-coated nanoparticles synthesized by hydrothermal precipitation. Fabrication costs for the latter nanomaterials are twenty times higher because of the finer control over final product characteristics, like size and shape uniformity and biocompatible coating (Augusto et al., 2020). The calculated material cost for co-precipitation was eighteen times smaller than in our process (Table 4), yet it represented 68% of direct operational costs (Supplementary Figures 4A,B). However, hydrothermal synthesis shows both a material cost (Table 4) and its relative participation on direct operational costs (Supplementary Figure 4) very similar to the bioprocesses. These results indicate that despite expenditures in feedstocks are very close to other processes, non-material costs are the main factors distinguishing bioprocess economics from chemical fabrication. This finding is further supported by the bigger proportion related to direct costs–54.9% for co-precipitation and 68.3% for hydrothermal–among total operational costs (Supplementary Figure 4A) in chemical processing. Accordingly, equipment costs in chemical syntheses are about 6–12 times lower than those in our process (Table 4). Thus, chemical synthesis presents a significantly lower operational cost. Another aspect to be considered in the techno-economic analysis is the energy consumption by the studied processes. BMN bioprocess shows a power consumption intermediary to co-precipitation and hydrothermal syntheses (Supplementary Table 10). As the latter is related to better control of nanoparticle characteristics, energy demand is directly correlated to the quality of the nanoproducts. In this way, BMN production is energy-efficient because even at consumption lower than a chemical process, bioproduction yields high-quality nanomagnets. Despite our product prices are higher than in chemical manufacturing, our MSP (US$ 21–120/g) still ranges significantly lower than commercial prices of most synthetic iron oxide nanoparticles of similar sizes (Figure 7 and Supplementary Table 11). Nanoparticles traded as “iron oxide nanopowders” are significantly cheaper, but often display poor size and shape distribution (refer to websites listed on Supplementary Table 11), similarly to those in Figure 1B. Bare magnetite nanoparticles prices are US$ 10,000–11,000/kg, values that can be 80–500 times our MSP. BMNs membrane displays a range of functional groups (e.g., ammine, phosphate, and carboxyl) that facilitates surface modification. Average prices for ammine-, PEG- and carboxyl-coated nanomagnets are approximately US$ 40,000, 32,000, and 100,000/kg. Hence, BMNs have a competitive potential for market entry in terms of commercial costing, even if process-related perturbations result in increases in MSP. The discrepancy between MSP and the market price of synthetic nanoparticles also allows an increase in sale prices, which decreases the investment payback time (Figure 5) and increases the return on investment in the project as well as the revenues generated.

**TABLE 4:** Comparison between production costs for biogenic and synthetic magnetic nanoparticles.

**Nanoparticle**	**Preparation method**	**Production costs (US$/kg Fe_3_O_4_)**	**Material costs (US$/kg Fe_3_O_4_)**	**Equipment costs (US$ millions)**	**References**
Biological-origin magnetic nanoparticles (BMNs)	Single stage	10,372	1,472	8,898	Present work
BMNs	Semicontinuous	11,169	1,477	12,888	Present work
Bare magnetite	Co-precipitation	210	78	1,036	Augusto et al., 2020
Carbon-coated magnetite	Hydrothermal	4,192	1,361	2,027	Augusto et al., 2020

Production of genetically engineered BMNs designed for specific applications have been successfully performed and might increase BMN competitivity in the nanotechnological market. Recently, the modification of both mineral characteristics (morphology and size) and surface coating can be modulated by genetic engineering (Furubayashi et al., 2020). BMNs expressing protein A on their surface were produced for the detection of pathogens and pollutants molecules (Xu et al., 2019). Applications proofs showed that, when bound to specific antibodies, the nanocomplex could be used for high-sensitivity detection of *Vibrio parahaemolyticus* (detection limit = 5 CFU/mL) and gentamicin (0.01 ng/mL). Costs calculated for their production in a 42-L fed-batch revealed the BMNs-protein A complexes incredibly inexpensive (US$ 0.067/mg) when compared to commercial immunomagnetic beads (US$ 3 or more).

Commercial catalogs of iron-oxide nanoparticles show some properties of their products: size dispersion, zeta potential (Zp), magnetization saturation (Ms), and purity (Supplementary Table 11). It is well-documented that BMNs show a narrow size distribution due to genetic-level controlled biomineralization. Because size and shape directly influence magnetic properties, like Ms (Mirabello et al., 2016), uniformity in those characteristics ensures reproducibility in applicability outcomes. Ms of BMNs is reportedly higher (61 emu/g at 290 K–Timko et al., 2009) than commercial nanomagnets (20–50 emu/g). In nanomagnetism, higher Ms values are related to better responsiveness to magnetic fields and higher heating properties in magnetically induced hyperthermia (Abenojar et al., 2016). Indeed, the heating capacity of BMNs has been examined and is compatible with hyperthermal treatments (Timko et al., 2009). Furthermore, BMNs display a very similar Zp (−38 to −25 mV–Geng et al., 2019; Xu et al., 2019) to carboxyl iron-oxide nanoparticles (−35 to −15 mV). This property ensures good colloidal stability of magnetic suspension due to electrostatic repulsion between nanoparticles (Bhattacharjee, 2016). BMNs are single-domain magnetic materials (Abreu et al., 2020b) and those repulsive interactions prevent particle aggregation, which could otherwise hinder applicability.

Although the process requires an entire downstream section dedicated to BMN recovery, the final nanoproduct retains its external membrane. Hence, additional coating procedures, often present in chemical production (Zhang et al., 2012; Augusto et al., 2020; Pinelli et al., 2020), are dispensed. The natural membrane envelope found in BMNs improves biocompatibility characteristics by reducing toxicity against human and animal cells and the environment (Revathy et al., 2017). The low affinity between phospholipids and iron oxide nanometric surfaces poses a technical hurdle for the artificial membrane coating of synthetic nanoparticles (Pinelli et al., 2020).

The effect and the fate of BMNs in the human organism is a primary source of concern regarding biomedical applications such as drug delivery and MRI contrasting. As with other bacterial-derived products, one great concern surrounding BMN biomedical utilization is the contamination with bacterial endotoxins, most notably LPS (World Health Organization (WHO), 2020). Given all known MTB are Gram-negative (Abreu et al., 2020b), residual LPS from cell lysis procedures might persist and invalidate use in healthcare. Nevertheless, laboratory-scale isolation procedures, like ultrasonic crushing and alkaline washing, greatly reduces endotoxin contamination in BMNs to levels compared to chemically synthesized iron oxide nanoparticles (Mandawala et al., 2017). Moreover, the MSC-based BMN extraction has shown efficiencies of up to 99.7% in the removal of cell debris (Rosenfeldt et al., 2020). Recently, a long-term (135 days) *in vivo* study on the biocompatibility of BMNs, showed that even at concentrations 10–50 times higher than previously tested for synthetic nanoparticles, tissue damage was negligible. This biocompatibility trend was maintained even in the liver and spleen where BMN concentration was greater (Nan et al., 2021). The same work also suggested that BMN clearance from the body occurred through biliary excretion, within 1 week from administration, and urinary excretion, up to 120 days (Nan et al., 2021). Given these elimination routes are already well-described for other drugs (Bardal et al., 2011), the pharmacological behavior of BMNs becomes more predictable.

The present work was elaborated in the year 2020 when the COVID-19 pandemic took place. Effects of the pandemic on the global economy may cast some uncertainty on market estimates reported here. However, the production of eco-friendly, bacterial-gestated multipurpose nanoparticles might constitute a valuable opportunity for the urgent sustainable recovery of the Latin American economy (León and Cárdenas, 2020). As an example, a team from Yachay Tech University, in Ecuador, developed a cheap and efficient poly-amino-ester for the extraction of RNA from SARS-Cov-2 preliminarily to PCR testing (Chacón-Torres et al., 2020). As discussed earlier, BMNs are effectively used in highly sensitive pathogen detection technologies through magnetically based cell/antigen concentration (Vargas et al., 2018; Xu et al., 2019). This relatively easy applicability, along with its low production prices and reproducible physical characteristics, makes BMNs obvious candidates for large-scale use in pandemic control purposes.

The techno-economic assessment of large-scale production of microbial nanomagnets gives a preliminary yet valuable understanding of how feasible the supplying of these promising materials for technological applications is. In the final analysis, the base production cost BMNs (US$ 10–11 thousand/kg Fe_3_O_4_) is 2.5–53 times higher than the chemical production of bare magnetite nanoparticles depending on the process. This difference is mainly because of indirect operating costs (76–79% of total process costs), which derive from higher equipment purchase costs (6–12 times higher) and labor for maintaining a bioprocess. Production costs and selling prices are significantly influenced by operational parameters (e.g., bacterial magnetite production) but only slightly altered by external economic factors, like material purchase prices. Still, the strong discrepancy between production costs and selling prices of coated artificial magnetite nanoparticles (US$ 11–40 thousand/g) enables BMNs to have economically attractive prices (US$ 21–120/g of MSP). Therefore, it is possible to sell BMNs at values higher than MSP to reduce investment payback time and maximize profits. Due to the superior characteristics of BMNs in relation to synthetic nanoparticles and cleaner production, the bioproduction costs are justifiably higher than chemical manufacturing. Considering the direct functionalization of BMNs due to their natural membrane and the possibility of customizing BMNs through genetic engineering, biogenic nanoparticles are applicable for diverse purposes. Moreover, the industrial production of BMNs might have improved yields either by genetic engineering of known strains of MTB or uncovering new culturable strains with more efficient metabolisms.

## Data Availability Statement

The original contributions presented in the study are included in the article/Supplementary Material, further inquiries can be directed to the corresponding author/s.

## Author Contributions

TC executed the process simulation and economic/sensitivity analyses and wrote most of the manuscript. RP contributed to the simulation and economic/sensitivity analysis. FA planned the study design, revised the data presented, wrote and edited the manuscript. All authors contributed to the article and approved the submitted version.

## Conflict of Interest

The authors declare that the research was conducted in the absence of any commercial or financial relationships that could be construed as a potential conflict of interest.

## Publisher’s Note

All claims expressed in this article are solely those of the authors and do not necessarily represent those of their affiliated organizations, or those of the publisher, the editors and the reviewers. Any product that may be evaluated in this article, or claim that may be made by its manufacturer, is not guaranteed or endorsed by the publisher.

## Supplementary Material

Supplementary Information Criteria used for modeling the downstream section.

Supplementary Figure 1 Number of registered nanotechnological products in Latin America categorized by country **(A)** and end-user sector **(B)**. Data from StatNano (2020).

Supplementary Figure 2 Magnetic separation column design.

Supplementary Figure 3 Sensitivity analyses showing effects of variations in economic **(A)** and bioprocess-related **(B)** parameters on unitary production costs and minimum selling prices (MSP) for the semicontinuous process when one of those individual parameters oscillates from the base-case (purple dashed vertical line). The results of variations in purchase price of two important feedstocks (lactic acid and urea) and fluctuations of the exchange ratio on the final production price are analyzed in panel **(A)**. The effects of specific cell growth rate of *Ms. gryphiswaldense* MSR-1 in fermentation tanks, as well as its magnetite production rate, are analyzed in panel **(B)**. The efficiency in BMN extraction by the MSCs and the plant annual throughput capacity, in terms of total produced magnetite, are also assessed in panel **(B)**. The purple dashed vertical line indicates base-case scenario whose parameters are described in Tables 1, 2.

Supplementary Figure 4 Operating costs composition breakdowns for the production of magnetic nanoparticles by synthetic chemical routes based on the data reported by Augusto et al. (2020). Direct and indirect cost contributions for co-precipitation and hydrothermal processes **(A)**. Direct operating costs breakdown showing cost types **(B)**. Material costs compositions from co-precipitation **(C)** and hydrothermal **(D)** processes.

Supplementary Table 1 Iron oxide nanoparticle demand estimation for the selected market.

Supplementary Table 2 Method used for plant cost calculations.

Supplementary Table 3 Method used for operating cost calculations.

Supplementary Table 4 Cost of raw material.

Supplementary Table 5 Cost of utilities.

Supplementary Table 6 Cost of labor, financing conditions and price indices.

Supplementary Table 7 Material factors and prices for MSC.

Supplementary Table 8 Purchase costs of equipment for single-stage fed batch.

Supplementary Table 9 Purchase costs of equipment for a semicontinuous process.

Supplementary Table 10 Comparison between approximate energy demands for biogenic and synthetic magnetic nanoparticles.

Supplementary Table 11 Comparison between minimum selling price of biogenic magnetite and commercial prices of synthetic iron oxide nanoparticles.
