# Supplementary material for: Why Does Not Nanotechnology Go Green? Bioprocess Simulation and Economics for Bacterial-Origin Magnetite Nanoparticles
Source: Front Microbiol. 2021 Aug 20;12:718232. doi: 10.3389/fmicb.2021.718232 (PMC8418543; doi:10.3389/fmicb.2021.718232)
Supplement: Supplementary Information — Criteria used for modeling the downstream section. [file Data_Sheet_1.docx]

Supplementary Material

# Supplementary Information - Downstream section design

Guo et al., (2011) designed a magnetic isolation system comprised of a high-pressure homogenizer, a magnetic separation column (MSC), an ultrasonic bath tank, and an electro-elution tank. Because of extreme energy consumption and heat release – and the consequent need for refrigeration – ultrasonic lysis is not adapted for industrial scales (de Carvalho, 2017). Thus, we have not considered this operation in our simulation. Another magnetic separation system has been developed by Rosenfeldt et al., (2020), where, like the previous one, high-pressure cell lysate goes through an MSC for BMNs enrichment. Nevertheless, the designs of MSCs in those two works show a crucial difference. While 5-mm carbon stells beads are used as the magnetizable matrix in the first work, the matrix in the latter is a ferromagnetic fiber. The matrix material seems to substantially influence BMNs separation. In the first case, 300 mg BMNs are recovered from 6 L culture – approximately 50 mg/L of final yield. Using a fibrous matrix, it was related that only 66.5% of adsorbed iron (in form of BMNs) is recovered after column percolation. Therefore, our simulated MSC matrix is composed of 2-mm stainless beads (Figure S2), assuming a presumably better BMNs recovery. Here, the bead diameter is reduced for a larger adsorbing surface. Additionally, the column wall material, aluminum, was chosen because it is a non-ferromagnetic metal. In Rosenfeldt et al., (2020), MSC elution is followed by an ultracentrifugation step in which BMNs are sedimented onto a sucrose cushion. In industry, ultracentrifugation is not usually used for large-scale separation (Harrison et al., 2015). Alternatively, we opted for a disk-stack centrifuge due to its versatility in separating small-dimension solids, high rotation speeds, and large-volume processing capacity (Talerton & Wakeman, 2006).

# Supplementary Figures

**Supplementary Figure 1.** Number of registered nanotechnological products in Latin America categorized by country (A) and end-user sector (B). Data from StatNano (2020).

**Supplementary Figure 2**. Magnetic separation column design.

**Supplementary Figure 3**. Sensitivity analyses showing effects of variations in economic (A) and bioprocess-related (B) parameters on unitary production costs and minimum selling prices (MSP) for the semicontinuous process. The purple dashed vertical line indicates base-case scenario.

**Supplementary Figure 4.** Operating costs composition breakdowns for the production of magnetic nanoparticles by synthetic chemical routes based on the data reported by Augusto et al (2020). Direct and indirect cost contributions for co-precipitation and hydrothermal processes (A). Direct operating costs breakdown showing cost types (B). Material costs compositions from co-precipitation (C) and hydrothermal (D) processes.

# Supplementary Tables

**Supplementary Table 1**. Iron oxide nanoparticle demand estimation for the selected market

| Parameter | Value | Reference |
| --- | --- | --- |
| International consumption (2019) | 3500 ton | Nano-Powder Factory (2020) |
| Demand increase for the period 2020-2022 | 12% | Nano-Powder Factory (2020) |
| Registered nanotechnological products - World | 8963 | Nano-Powder Factory (2020) |
| Share of nanotechnological products in Latin America | 2.4% | Nano-Powder Factory (2020) |
| Share of products in the biomedical and environmental sectors | 4.6% | Nano-Powder Factory (2020) |
| Estimated annual demand in Latin America for the selected sectors | 640 kg | estimated |

**Supplementary Table 2**. Method used for plant cost calculations.

| Parameter | Method of calculation |
| --- | --- |
| **Total plant direct cost (TPDC)** | TPDC = PC + C*_installation_* + C*_piping_* + C*_instrumentation_* + C*_insulation_* + C*_electrical_* + C*_buildings_* + C*_yard_* + C*_auxiliary_* |
| Equipment purchase cost (PC) | Simulated based on process sizing and cost indexes |
| Instalation (C*_installation_*) | = PC × 0.30 (for fermentation section and seed train)  = PC × 0.50 (for nanoparticle extraction section) |
| Process piping (C*_piping_*) | = PC × 0.35 |
| Instrumentation (C*_instrumentation_*) | = PC × 0.40 |
| Insulation (C*_insulation_*) | = PC × 0.03 |
| Electrical facilities (C*_electrical_*) | = PC × 0.10 |
| Buildings (C*_buildings_*) | = PC × 0.45 |
| Yard improvement (C*_yard_*) | = PC × 0.15 |
| Auxiliary facilities (C*_auxiliary_*) | = PC × 0.40 |
| **Total plant indirect cost (TPIC)** | PIC = C*_engineering_* + C*_construction_* |
| Engineering (C*_engineering_*) | = TPDC × 0.25 |
| Construction (C*_construction_*) | = TPDC × 0.35 |
| **Total plant cost (TPC)** | TPC = TPDC + TPIC |
| Contractor’s fee | = TPC × 0.05 |
| Contingency | = TPC × 0.10 |
| **Direct fixed capital (DFC)** | DFC = TPC + Contractor’s fee + Contingency |
| Working capital | = DFC × 0.003 |
| Start-up validation | = DFC × 0.05 |
| **Capital Investment** | **= DFC + Working capital + Start-up validation** |

**Supplementary Table 3**. Method used for operating cost calculations.

| Cost item | Type | Method of calculation |
| --- | --- | --- |
| Raw materials | Direct | Simulated based on prices listed on Table S2 |
| Labor | Direct | = Basic rate (Table S4) × 1.3 |
| Consumables | Direct | Simulated based on prices listed on Table S5 |
| Laboratory / Quality control | Direct | = Labor × 0.15 |
| Waste treatment and disposal | Direct | Simulated based on prices listed on Table S3 |
| Utilities | Direct | Simulated based on prices listed on Table S3 |
| Facility-related | Indirect | Equipment-dependent |
| Miscellaneous | Indirect | = 0.08 × TPC |

**Supplementary Table 4**. Cost of raw material

| Material | US$/kg | Source |
| --- | --- | --- |
| Lactic acid | 3.00 | Adicel.com.br |
| Sodium lactate | 1.50 | Molbase.com |
| NH_4_OH | 0.45 | Echemi.com |
| NH_4_Cl | 0.093 | Echemi.com |
| Yeast extract | 2.5 | Molbase.com |
| FeCl_3_ | 0.49 | Echemi.com |
| MgSO_4_ | 0.086 | Echemi.com |
| NaNO_3_ | 1.25 | Echemi.com |
| Mineral elixir^a^ | 0.32 | Echemi.com |
| Na_2_HPO_4_ | 5.06 | Molbase.com |
| KH_2_PO_4_ | 1.13 | Echemi.com |
| NaCl | 0.335 | Echemi.com |
| KCl | 0.269 | Echemi.com |
| Urea | 0.25 | Echemi.com |
| Sucrose | 0.26 | Mfrural.com.br |
| NaOH | 0.01 | Molbase.com |
| H_3_PO_4_ | 0.03 | Echemi.com |
| Process water | 5.91^b^ | de Andrade (2014) |
| ^a^ calculated from individual components as in Wolin, Wolin and Wolfe (1963) | | |
| ^b^ price in US$/m³, based on the process of BioManguinhos, Rio de Janeiro, Brazil | | |

**Supplementary Table 5**. Cost of utilities

| Material | Price (US$) | Source |
| --- | --- | --- |
| Potable water | 3.27/m³ | de Andrade (2014) |
| Steam^a^ | 16.82/MT | Ruediger (2014) |
| Electricity | 86.11/MWh | ANEEL (2020) |
| Effluent treatment^b^ | 0.63/m³ | Dalri-Cecato et al. (2019) |
| ^a^ for steam generated on natural gas | | |
| ^b^ for a membrane bioreactor treatment | | |

**Supplementary Table 6**. Cost of labor, financing conditions and price indices

| *Labour* | | |
| --- | --- | --- |
| Average salary for pharma-chemical sector | R$ 52 746.57/year | IBGE (2019) |
| *Financing* | | |
| Debt | up to 80% of project | Finep (2020) |
| Loan period | up to 10 years | Finep (2020) |
| Loan interest rate | 8.42% | Calculated Finep rate for innovative medium-sized industrial projects |
| *Price indices* | | |
| CE Plant Cost Index | 607.5 (for 2019) | Chemical Engineering (2020) |
| Producer Price Index (Brazil) | 116.29 (for 2020) | IBGE (2020) |
| Dollar/Real exchange rate | 5.20 (as of June, 2020) | Banco Central do Brasil (2020) |

**Supplementary Table 7**. Material factors and prices for magnetic separation columns

| Item | Material factor or price | Reference |
| --- | --- | --- |
| Carbon steel | 1.0 | Towler & Sinnott (2013) |
| Aluminium and bronze | 1.07 | Towler & Sinnott (2013) |
| Aluminium plus neodymium plates | 1.27 | Calculated from Towler & Sinnott (2013) and neodymium magnet prices |
| Stainless steel beads (2 mm) | US$ 24/kg | mercadolivre.com.br |
| Neodymium plates | US$ 0.44/cm³ | mercadolivre.com.br |

**Supplementary Table 8**. Purchase costs of equipment for single-stage fed batch

| Section / Equipment ID | Description | Purchase cost (US$) |
| --- | --- | --- |
|  |  |  |
| *Inoculum train* | | |
|  |  |  |
| SFR-102 | Seed Fermentor (24.7 L) | 588 000 |
| SFR-103 | Seed Fermentor (247 L) | 630 000 |
| SFR-104 | Seed Fermentor (2470 L) | 872 000 |
|  |  |  |
| *Fermentation* | | |
|  |  |  |
| BR-101 | Bioreactor (29 m³) | 2 724 000 |
| V-10 | Blending Tank (2.8 m³) | 263 000 |
| V-102 | Blending Tank (20 m³) | 348 000 |
| ST-101 | Heat Sterilizer (Feed medium) | 489 000 |
| ST-102 | Heat Sterilizer (Fermentation medium) | 574 000 |
| G-101 | Centrifugal Air Compressor | 87 000 |
| AF-101 | Air Filter | 9 000 |
| HG-101 | High-pressure homogenizer | 125 000 |
|  |  |  |
| *Nanoparticle Extraction* | | |
|  |  |  |
| MIC-102 | MS Column (725 L) | 100 000 |
| MIC-101 | MS Column (2250 L) | 177 000 |
| DS-101 | Disk-Stack Centrifuge | 133 000 |
|  |  |  |
| *Whole process* | | |
|  |  |  |
| Not tagged | Pumping, inoculum media sterilizers, etc. | 1 780 000 |
|  |  |  |
|  | TOTAL | 8 898 000 |

**Supplementary Table 9**. Purchase costs of equipment for a semicontinuous process

| Section / Equipment ID | Description | Purchase cost (US$) |
| --- | --- | --- |
|  |  |  |
| *Inoculum train* | | |
|  |  |  |
| SFR-102 | Seed Fermentor (36.1 L) | 588 000 |
| SFR-103 | Seed Fermentor (361 L) | 665 000 |
| SFR-104 | Seed Fermentor (3610 L) | 920 000 |
|  |  |  |
| *Fermentation* | | |
|  |  |  |
| BR-101 | Bioreactor (22.6 m³ x 2) | 4 924 000 |
| V-10 | Blending Tank (3.9 m³ x 2) | 552 000 |
| V-102 | Blending Tank (32 m³) | 390 000 |
| ST-101 | Heat Sterilizer (Feed medium) | 673 000 |
| ST-102 | Heat Sterilizer (Fermentation medium) (x 2) | 1 175 000 |
| G-101 | Centrifugal Air Compressor | 87 000 |
| AF-101 | Air Filter | 9 000 |
| HG-101 | High-pressure homogenizer (x 3) | 354 000 |
|  |  |  |
| *Nanoparticle Extraction* | | |
|  |  |  |
| MIC-102 | MS Column (1334 L) | 136 000 |
| MIC-101 | MS Column (4140 L) | 240 000 |
| DS-101 | Disk-Stack Centrifuge | 133 000 |
|  |  |  |
| *Whole process* | | |
|  |  |  |
| Not tagged | Pumping, inoculum media sterilizers, etc. | 1 990 000 |
|  |  |  |
|  | TOTAL | 12 888 000 |

**Supplementary Table 10**. Comparison between approximate energy demands for biogenic and synthetic magnetic nanoparticles.

| Nanoparticle | Preparation method | Power consumption (kWh/kg Fe_3_O_4_) | Energy costs (US$/kg Fe_3_O_4_) | % Fabrication costs | Reference |
| --- | --- | --- | --- | --- | --- |
| BMNs | Single stage | 1334.14 | 112.47 | 4.5 | Present work |
| BMNs | Semicontinuous | 1795.48 | 151.36 | 6.3 | Present work |
| Bare magnetite | Co-precipitation | N/A | 42.00 | 20 | Augusto et al., 2020 |
| Carbon-coated magnetite | Hydrothermal | N/A | 838.40 | 20 | Augusto et al., 2020 |

**Supplementary Table 11**. Comparison between minimum selling price of biogenic magnetite and commercial prices of synthetic iron oxide nanoparticles

| Nanoparticle | Size (nm) | Price (US$/g) | Available description |
| --- | --- | --- | --- |
|  |  |  |  |
| Magnetosomes | 33 | 21 – 120 (MSP) | see text |
|  |  |  |  |
| *Ocean Nanotech* (https://www.oceannanotech.com/products/) | | | |
|  |  |  |  |
| Amine iron oxide nanopaticles | 30 | 31 960 | ZP = +5 to +15 mV |
| Carboxyl iron oxide nanoparticles | 30 | 15 980 | ZP = -35 to -15 mV |
| PEG iron oxide nanoparticles | 30 | 31 960 | ZP = -10 to 0 mV |
|  |  |  |  |
| *Cytodiagnostics* (https://www.cytodiagnostics.com/) | | | |
|  |  |  |  |
| Iron oxide magnetic nanoparticles | 20 ± 3 | 11 000 | Ms > 20 emu/g |
|  |  |  |  |
| *Millipore-Sigma* (https://www.sigmaaldrich.com/) | | | |
|  |  |  |  |
| Iron (II,III) oxide nanopowder | 50-100 | 1.30 | 97% purity |
| Iron oxide (II,III), nanoparticles | 30 | 10 480 | - |
| Iron oxide (II,III), nanoparticles, amine functionalized | 30 | 48 600 | Ms > 45 emu/g |
| Iron oxide (II,III), nanoparticles, carboxilic functionalized | 30 | 255 000 | Ms > 45 emu/g |
| Iron oxide (II,III), nanoparticles, PEG | 30 | 34 800 | Ms > 48 emu/g |
|  |  |  |  |
| *SkySpring Nanomaterials Inc.* (https://ssnano.com/) | | | |
|  |  |  |  |
| Iron Oxide Nanopowder | 20-30 | 0.34 | 98% purity |

# References

Guo, F., Liu, Y., Chen, Y., Tang, T., Jiang, W., Li, Y., and Li, J. (2011). A novel rapid and continuous procedure for large-scale purification of magnetosomes from *Magnetospirillum gryphiswaldens*e. Appl. Microbiol. Biotechnol. 90:4. doi: 10.1007/s00253-011-3189-3

Rosenfeldt, S., Mickoleit, F., Jörke, C., Clement, J.H., Markert, S., Jérôme, V., Schwarzinger, S., Freitag, R., Schüler, D., Uebe, R., and Schenk, A.S. (2020). Towards standardized purification of bacterial magnetic nanoparticles for future in vivo applications. Acta Biomater [Preprint]. Available at: <https://doi.org/10.1016/j.actbio.2020.07.042>

Abreu, F., Morillo, V., Trubitsyn, D., and Bazylinski, D.A. (2020b). “Magnetotaxis in Prokaryotes.” in eLS. (Chichester: John Wiley & Sons, Ltd). doi: 10.1002/9780470015902.a0000397.pub3

Harrison, R.G., Todd, P., Rudge, S.R., and Petrides, D.P. (2015). Bioseparations science and engineering. New York: Oxford University Press.

StatNano. (2020). Nanotechnology Products Database. StatNano Publications.

Nano-Powder Factory. (2020). Research of the world market of nanopowders. <https://eednano.com/> [Accessed March 24, 2020]

De Carvalho, J.C., Medeiros, A.B.P., Letti, L.A.J., Kirnev, P.C.S., and Soccol, C.R. (2017). “Cell Disruption and Isolation of Intracellular Products.” in Current Developments in Biotechnology and Bioengineering: Production, Isolation and Purification of Industrial Products, ed. A. Pandey (Amsterdam: Elsevier B. V.)

Tarleton, T., and Wakeman, R. (2006). Solid/Liquid Separation: Equipment Selection and Process Design. Oxford: Elsevier Science.

De Andrade, B. (2014) Reuso de efluentes industriais gerados durante a produção de água purificada na Central de Tratamento de Água do Centro Tecnológico de Vacinas de BioManguinhos/FIOCRUZ. [dissertation/master’s thesis]. [Rio de Janeiro, Brazil]: Fundação Oswaldo Cruz.

Dalri-Cecato, L., Battistelli, A.A., Schneider, E.E., Hassemer, M.E.N., and Lapolli, F.R. (2019). Operating cost assessment of a membrane bioreactor. REVISTA DAE. 217:1778. doi: 10.4322/dae.2019.025

Towler, G., and Sinnott, R. (2013). Chemical Engineering Design. Boston: Butterworth-Heinemann

Ruediger, R. (2014). Uso Racional do Vapor na Indústria. <https://fiesc.com.br/sites/default/files/inline-files/PALESTRA%20BERMO.pdf> [Access March 24, 2020]

ANEEL – Agência Nacional de Energia Elétrica. (2020). Tarifas médias por classe de consumo. https://www.aneel.gov.br/dados/tarifas [Access March 24, 2020]

IBGE – Instituto Brasileiro de Geografia e Estatística. (2019). Emprego, salário e encargos das empresas industriais com 30 ou mais pessoas ocupadas, segundo as divisões, os grupos e as classes de atividades – Brasil. <https://sidra.ibge.gov.br/tabela/7241> [Access March 24 2020]

IBGE – Instituto Brasileiro de Geografia e Estatística. (2020). Índice de Preços ao Produtor - Indústrias Extrativas e de Transformação – IPP. <https://www.ibge.gov.br/estatisticas/economicas/precos-e-custos/9282-indice-de-precos-ao-produtor-industrias-extrativas-e-de-transformacao.html> [Access September, 1, 2020]

FINEP – Financiadora de Estudos e Projetos. (2020). Condições Operacionais. <http://www.finep.gov.br/images/a-finep/Condi%C3%A7oes_Operacionais/CondicoesOperacionais.pdf> [Access September 1, 2020]

Chemical Engineering. (2020). 2019 Chemical Engineering Plant Cost Index Annual Average. <https://www.chemengonline.com/2019-chemical-engineering-plant-cost-index-annual-average/> [Access September 1, 2020]

Banco Central do Brasil. (2020). Cotações e boletins. <https://www.bcb.gov.br/estabilidadefinanceira/historicocotacoes> [Access September 1, 2020]

**
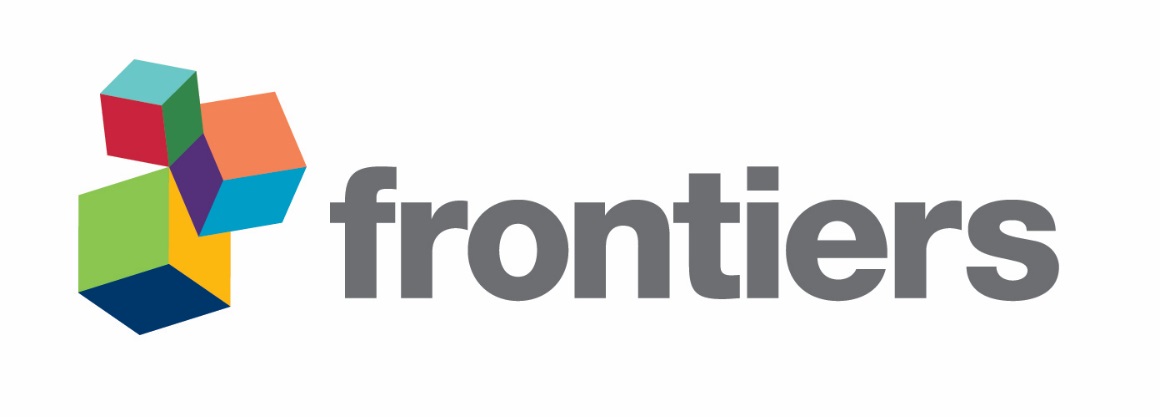
**
