# Supplementary figures and images for: Why Does Not Nanotechnology Go Green? Bioprocess Simulation and Economics for Bacterial-Origin Magnetite Nanoparticles
Source: Front Microbiol. 2021 Aug 20;12:718232. doi: 10.3389/fmicb.2021.718232 (PMC8418543; doi:10.3389/fmicb.2021.718232)

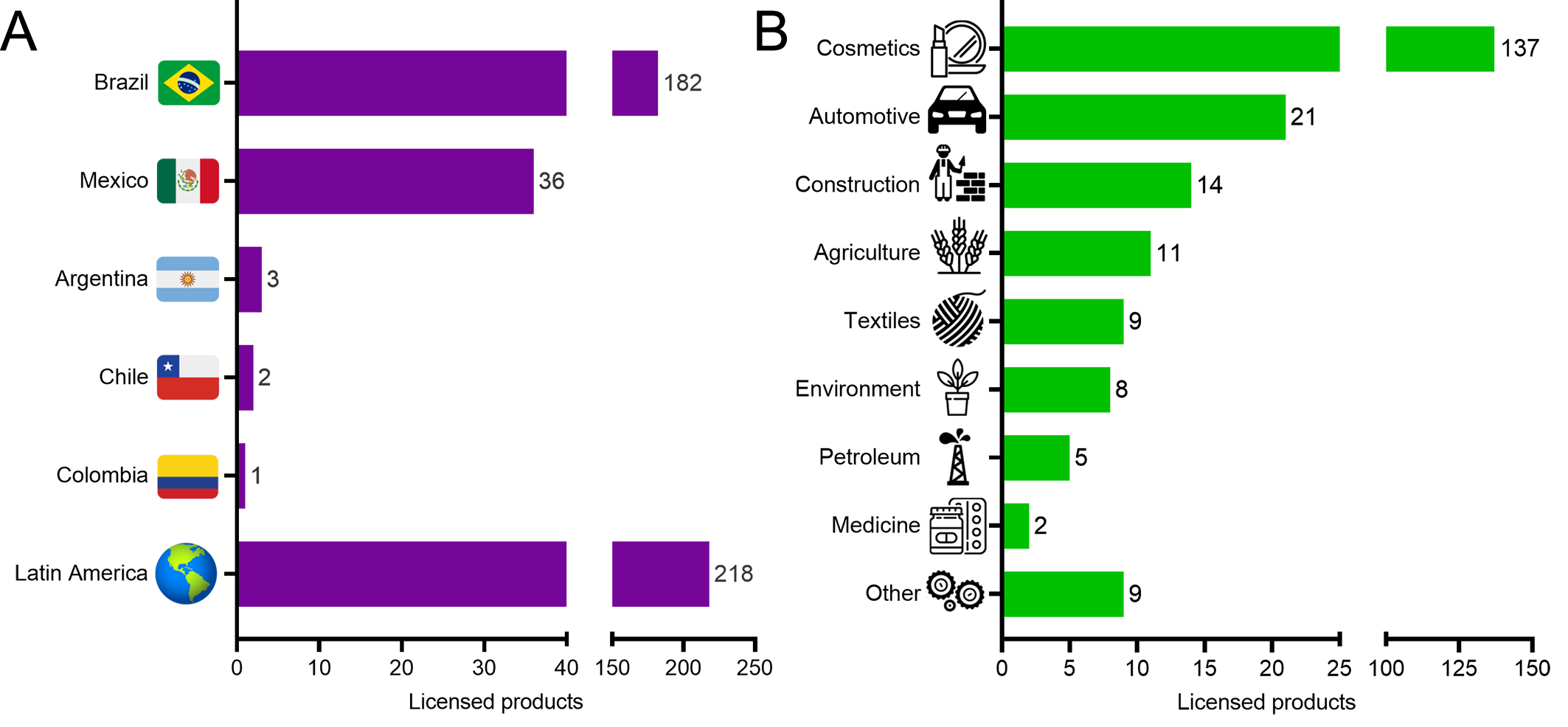

Supplement: Supplementary Figure 1 — Number of registered nanotechnological products in Latin America categorized by country (A) and end-user sector (B). Data from StatNano (2020). [file Image_1.TIF]

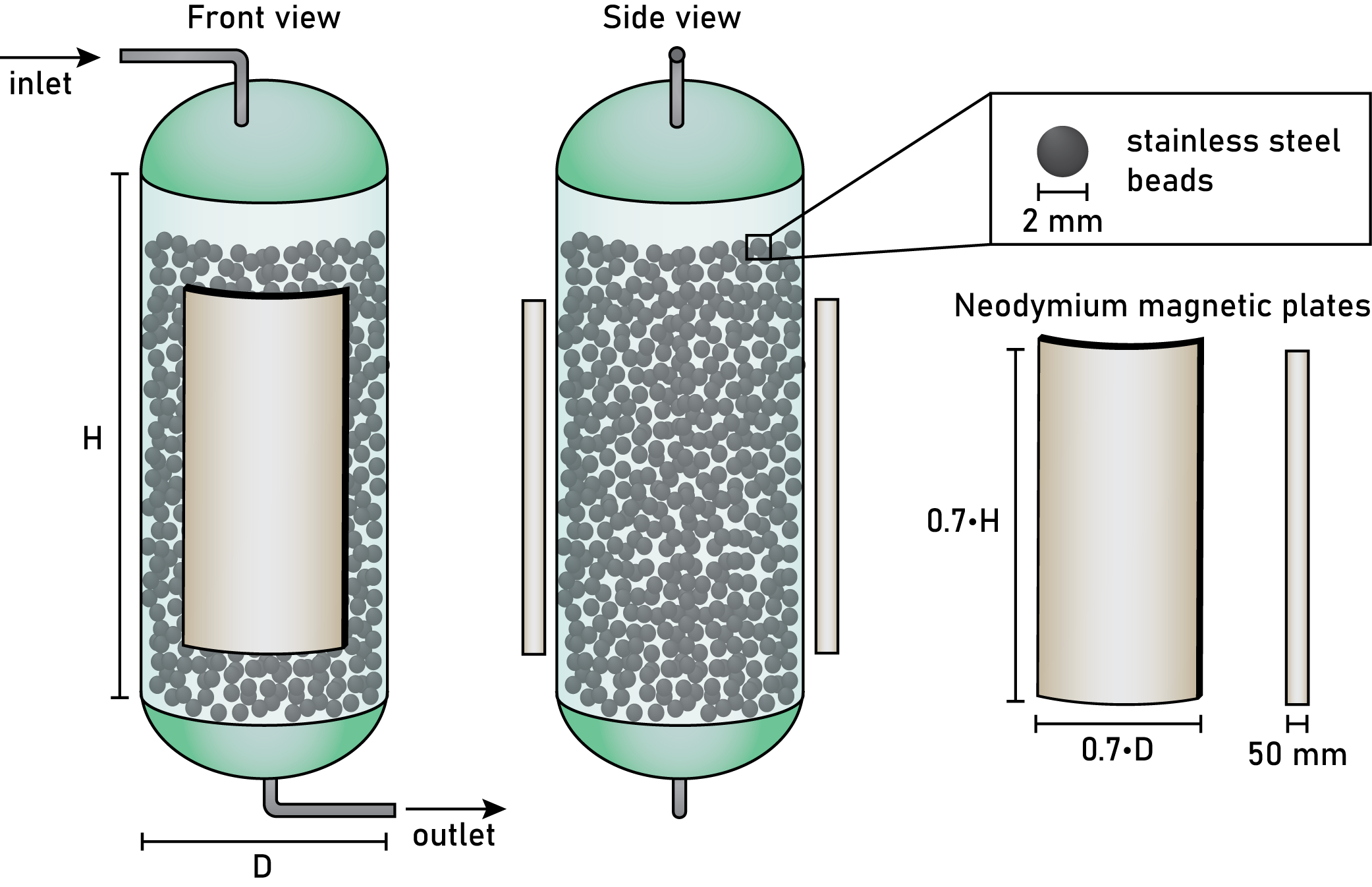

Supplement: Supplementary Figure 2 — Magnetic separation column design. [file Image_2.TIF]

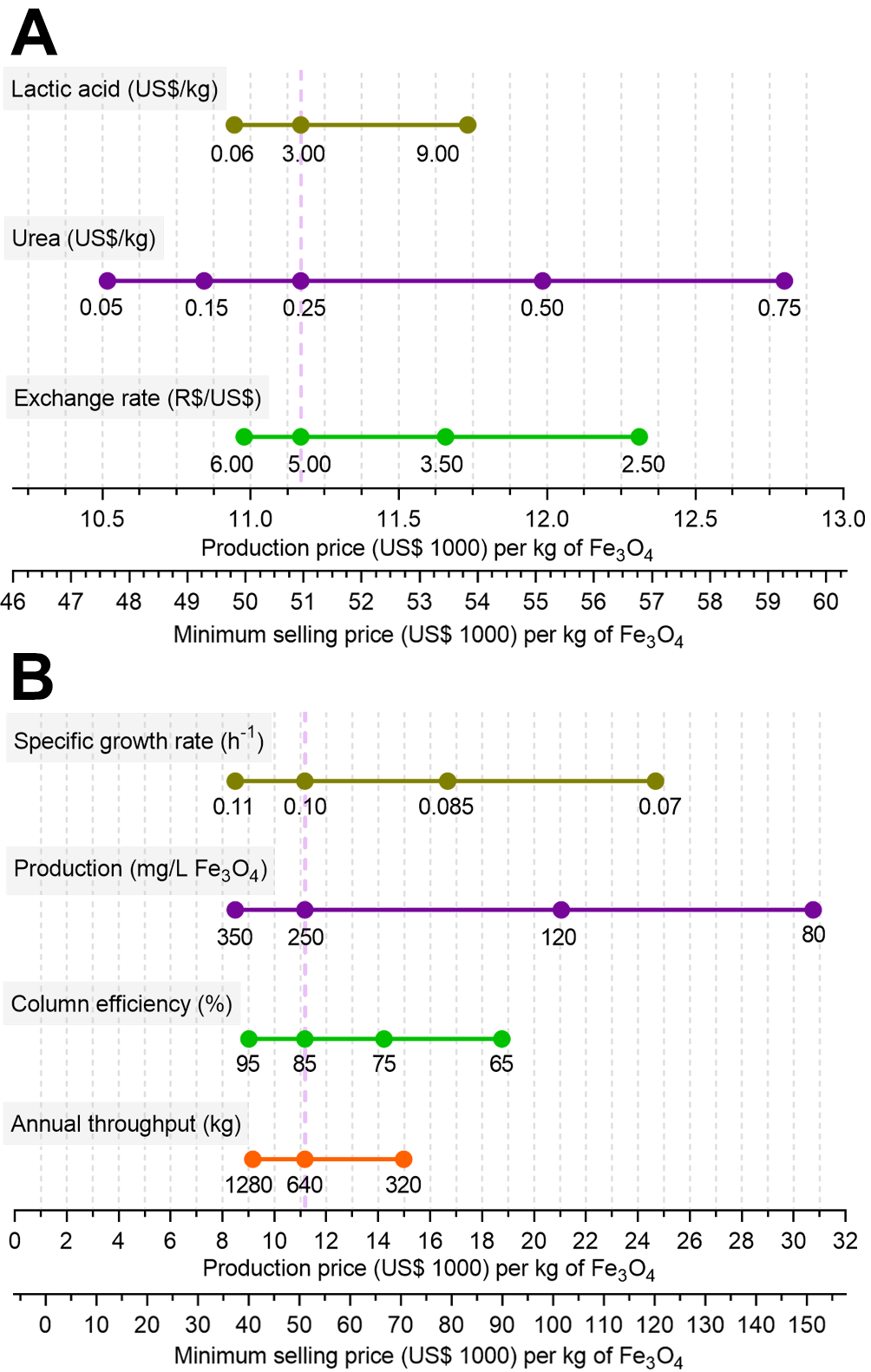

Supplement: Supplementary Figure 3 — Sensitivity analyses showing effects of variations in economic (A) and bioprocess-related (B) parameters on unitary production costs and minimum selling prices (MSP) for the semicontinuous process when one of those individual parameters oscillates from the base-case (purple dashed vertical line). The results of variations in purchase price of two important feedstocks (lactic acid and urea) and fluctuations of the exchange ratio on the final production price are analyzed in panel (A). The effects of specific cell growth rate of Ms. gryphiswaldense MSR-1 in fermentation tanks, as well as its magnetite production rate, are analyzed in panel (B). The efficiency in BMN extraction by the MSCs and the plant annual throughput capacity, in terms of total produced magnetite, are also assessed in panel (B). The purple dashed vertical line indicates base-case scenario whose parameters are described in Tables 1, 2. [file Image_3.TIF]

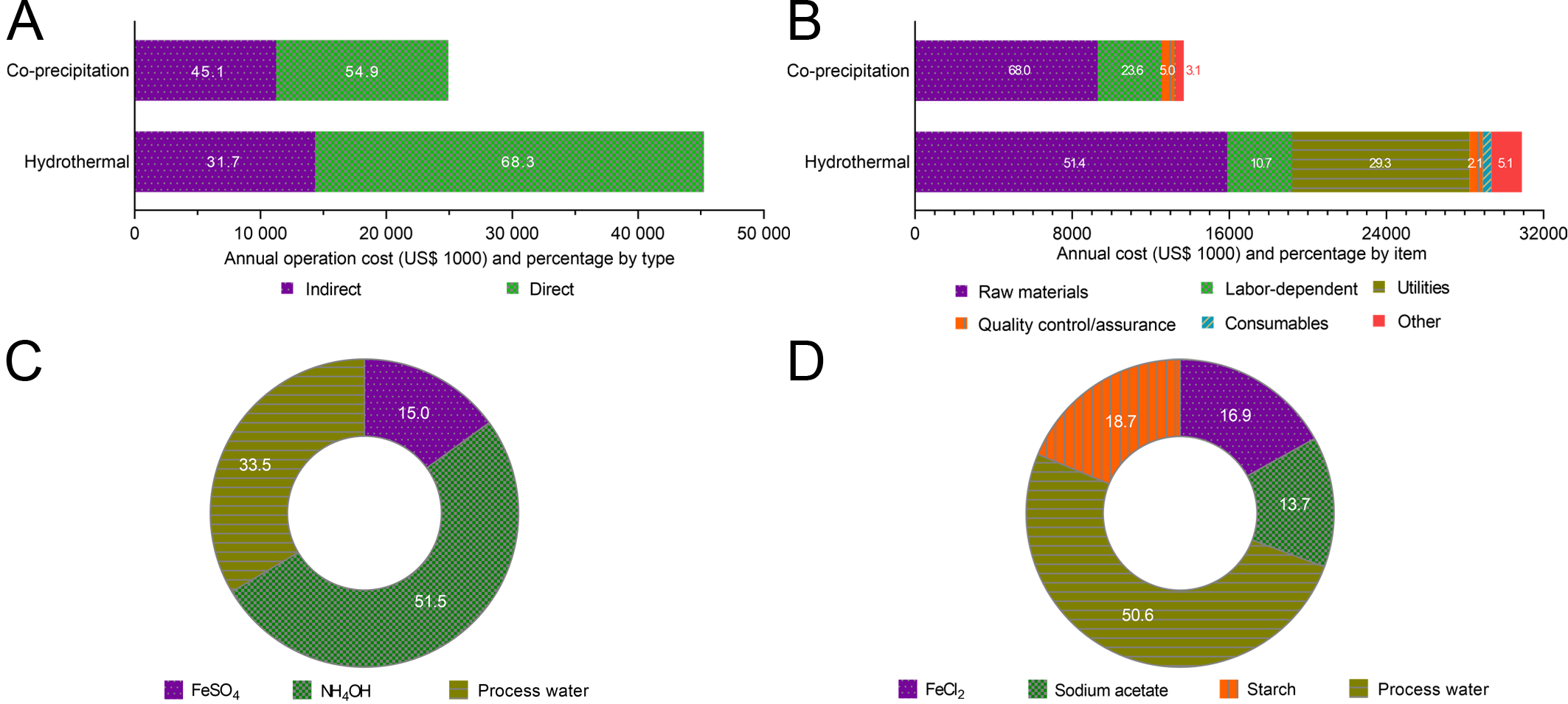

Supplement: Supplementary Figure 4 — Operating costs composition breakdowns for the production of magnetic nanoparticles by synthetic chemical routes based on the data reported by Augusto et al. (2020). Direct and indirect cost contributions for co-precipitation and hydrothermal processes (A). Direct operating costs breakdown showing cost types (B). Material costs compositions from co-precipitation (C) and hydrothermal (D) processes. [file Image_4.TIF]
